# Rachel Challice's Vow

**Published:** 1888-03-24

**Authors:** Lina Nevill

**Affiliations:** Author of "A Future on Trust," "Juno," &c.


### Rachel Challice's Yow.

By Lina Nevill, Author of "A Future on Trust," "Juno," &c.

### Chapter XV.—Lost.

"Oh! star of strength, I see thee stand
And smile upon my pain;
Thou beckonest with thy mailed hand,
And I am strong again."—*Longfellow.*

All through that long September night Rachel Challice watched and waited. Watched the struggles of a dying man fighting for life—waited the final issue of that strife.

The Angel of Death came flying into that poor cottage home, and hovered on poised wings above the bed where the sick man lay.

Death, in all its many aspects, was no stranger to Rachel. She had watched many lives ebbing away, till they lost themselves in the wide unfathomable sea of eternity. She had no fear, only always that awe which the sight of a departing spirit must generally inspire, when it goes out alone on its journey towards the great debateable land that lies beyond the horizon of our world, and we feel that here earthly power has reached its limit—that another and a stronger ruler steps in, and says in unanswerable tones: "Hitherto shalt thou come, and no further."

Rachel would not disturb Esther, who slept heavily in the adjoining room, where the two children also lay in the calm, souned sleep of childhood, unconscious of what was passing so nar them .

When the final struggle was near, then Rachel would call her sister; and, so far, David had not asked for his wife. He seemed perfectly happy and contented to have Rachel; a sort of instinct told him that she was to be depended upon. In the stillness of the dark night hours the gurgle of the rippling Glasslyn came softly and soothingly on Rachel's ear. The singing of the beautiful river seemed to tell her that someone else besides herself was keeping vigil during the long hours of that lonely night.

Outside, the autumn wind was sighing among the mountains. And they stood—mighty armies of mist-veiled, shrouded giants—in long lines of undulating ridges and sharp spiral peaks, waiting for the sun to break through the purple clouds in the east, and with his greeting kiss chase away the black shadows and sombre coverings of night.

So the hours wore on—the hours—numbered to David Stephens—of the last earthly night he should see.

When darkness should next cover the wild Welsh country there would be one life less in the world; one more empty place; one other spirit gone on a long journey to a distant land.

Suddenly he began to speak.

"Rachel," he said, "are you there; are you alone?"

"Yes, David, I am here. Esther is sleeping. Shall I call her?"

"No, not yet," he replied in his weak voice. "Come close to me, Rachel; I want to speak to you. Rachel, you know I am going to die?"

She knelt down by his bedside, so as to be quite near to him, and that there might be no chance of rousing Esther with their talk. Sleep was good for the poor, weak, worn-out wife, and there was no actual necessity for wakening her at present.

"Yes, David," said Rachel softly," I do know it. But you are not afraid?"

"No," he answered restlessly; "not more so than most, I suppose. I don't want to live, except for poor Esther's sake. We have not been very happy together, perhaps I did not make enough allowance for her. But you will see after her and my little ones when I am gone, won't you, Rachel?"

Once again Rachel Challice had solemnly to vow to a dying fellow-creature that her sister's welfare should be her first care. That she would shield and protect the girl who was so unfit to fight her own battle with the world. That, as before, she would place herself last and Esther first; and make herself of no account compared to her sister.

Vividly before her mind rose up the vision of that other death-bed, when alone she had watched a spirit leave a worn-out frame, and when such a sight had been new and terrible to her. She pictured the hard, cold, old woman, who had died without any love, or one kind word or thought for the girl watching so devotedly by her side; while her whole failing strength was spent in tender provision for the grandchild who was thoughtlessly amusing herself, and who had never striven to repay one tithe of the love lavished upon her.

That first watch of Rachel's had been a thankless task, this second one was a labour of love.

Though an impenetrable barrier reared its strong unbending wall between David and herself, and they both knew and realized that the division was there; she felt instinctively that a love and sympathy, pure and strong, and tried in the cleansing fires of adversity, existed between them, and that all former coldness and misunderstanding had been swept away.

Esther was his wife—neither for one moment forgot that—and Rachel was his friend and sister.

"Thank you, Rachel," he said in a tone of relief, as she assured him that the charge of Esther and the children should be her chief care. "I can die easy now. I know you will do better for them than anyone else. Father has been hard on us, you know. It was partly my fault, but I am sorry for it."

He seemed tired with so much talking, and lay still for a little while. Then he began again.

"Rachel, why would you not marry me when I asked you? I've often thought about it; and I never could make up my mind that you didn't love me at one time."

Her pale face became suddenly crimson, and she hesitated to reply. Then, with eternity full before them, with earth and its affairs far in the past, Rachel resolved to tell David the truth.

She felt—whether it were weak and wrong on her part, or no—that she could not let him die misjudging her. She was only a woman after all; and few women care to be doubted by the person they love best in the world, though pride often holds them back from declaring their motives openly.

"I loved you; but I would not marry you, David," she said steadily, "because I wished you to marry Esther. I thought it would be for her true happiness to become your wife. And I had promised Granny to give up everything for her."

She stated the fact simply, not with any view towards making her conduct appear more generous. It was more as an excuse for having done an action which she was not sure was right, but about which she had been given no choice.

"I was strong and hard, and able to fight the world alone, David. But poor little Esther was different; she was so pretty, and weak, and easily led. She often used to tell me she loved you. So I determined, somehow or other, to make you marry her."

"And you never thought of me, Rachel?" he said a little quaintly.

"I knew you would be happy with Esther. You had always seemed to love her at first. I could not believe you cared so much for me that it would make any real difference; it was a long time before I could persuade myself you cared for me at all. Nobody did, you know," she wound up pathetically; and yet there was no straining after effect in her words. She was only rehearsing what she knew to be plain unvarnished facts.

"I did care for you, Rachel," said David slowly.

"But you were just as happy to take Esther. When you married her I thought I had been quite right in all that I had done, and that after all you were the two made for each other."

"You thought yourself very wise, my dear, and tried to manage too much. Well, for poor Esther's sake, I won't say that I wish things had been different. I've been as much to blame as her. I knew she was giddy and foolish, and I ought to have had more patience, and tried to teach her better ways. If father hadn't been so dead against her things might have gone better, too. He likes you, Rachel. He would rather I had married you; he'd have given in to me then in time. Go to him when I'm dead, Rachel, and ask him to be kind to the children for my sake. He may do it for you. And tell him I am sorry for having gone against his wishes; and I'd like to have seen him to say good-bye, but I haven't time for that now."

Every moment his voice was growing weaker. The last sands of life were racing fast through the nearly empty hourglass.

Away beyond the eastern range of hills the sun was raising his broad golden face, thrusting aside the curtain of purple clouds that hung over the horizon, and decking the heavens with a glory of crimson and amber radiance.

The fresh morning breeze rippled the smooth surface of Dinas lake, where the salmon were leaping. And the sheep-bells were making merry tinkling music as the flocks wandered up and down over the mountain sides.

A new day had begun on the earth. A day of sunshine and joy, and of glowing autumn-decked beauty. A day that would take no heed to the life hovering on the very margin of the dark river that separates the hosts of the living from the dead. The day would run its allotted course of hours. Then, when night came, in its turn it would fade and die, and be thrust into that mighty store-house, where lie in vast half-forgotten heaps the dead days, weeks, months, and years of the past.

A gleam of the new day struggled through the cottage window, and the pale light fell across the bed where David lay almost wrapped in the encircling wings of Azrael, the dread angel.

"It has nearly come," whispered David, in faint, broken tones. "Kiss me once, Rachel, before I die."

With her eyes full of blinding tears, she bent down her face, and laid her lips on his, in one last, long farewell.

He was the only man she had ever loved, or would love so long as life lived in her. No wonder she felt the anguish of the parting almost more than she could bear.

"Now call Esther."

She roused her sleeping sister, and brought her into the chamber of death.

And, as the golden sun shook himself free from the circling clouds, and rose high in the blue morning heavens—when the busy hum of life began to be heard in Beth-gelert village—the angel, who had been waiting all night, went away. But not empty-handed; for when he left he carried with him the soul of David Stephens.

### Chapter XVI.—Too Late.

"Too late for the rose the evening rain—
Too late for the lamb the shepherd's pain—
Too late for the plea when the doom hath been spoke!
Too late the balm when the heart is broke."
—*Whyte Melville.*

An early visitor came to the cottage.

Soon after eight o'clock, the small, spare form of Elias Stephens, in his brown greatcoat and wideawake hat, came tramping down the road from Aberelwyn; and on he came, straight up to his son's house.

He took no notice of the greetings of the people he passed, beyond a short nod now and again. People watched him inquisitively as he walked up to the cottage, and peeped out at their doors to see if he would go in.

The quarrel between the father and son, and the cause of it had been common talk and property; and people were anxious to see, now that David was dead, how the old man meant to behave.

He looked up at the windows as he came near the house, and shut his thin lips more tightly as he saw the closed blinds. "What's all this?" he said sharply to Rachel, who had opened the door to him.

They stood in the dirty, empty kitchen. Esther was lying down upstairs and trying to keep the children quiet, who were fretting and whining to get up and have their breakfast. Rachel had been just beginning to dress little Essie when old Stephens' knock had come at the door.

"What does it all mean?" he said. "Folks are saying David is dead; that's what that fool Martha said when she gave me my breakfast. It can't be true. Why, he was at his work at the mill not more than ten days ago."

"It is true, Mr. Stephens," replied Rachel, the tears starting to her eyes. "David died two hours ago."

The girl looked very worn and weary; she had scarcely eaten anything since her first arrival, and the sorrowful night's watch had told greatly upon her. There was no colour left in her cheeks, her face looked pinched and thin, great black shadows circled round her large grey eyes, and tears had made them dull and expressionless.

Elias, with his keen glance, noted the difference between this pallid, worn-out young woman and the beautiful, active girl he had seen the night before, and he felt touched, and sorry for Rachel. He liked her all the more that sorrow for the death of his son should have wrought this change.

Elias had always respected Rachel; her cold, reserved ways had been to his taste. Had it been necessary that David should have married somewhat beneath him in point of position and money, no girl would have been more welcome as a daughter-in-law than Rachel Challice, though he had never in any way shown people his partiality for her.

"Why didn't you send for me?" he asked sharply. "A son should not be allowed to die without his father hearing of it. You should have known better than to allow such a thing, Rachel Challice."

"How should I know that you would care to hear?" she retorted. "You have taken no notice of your son for more than three years, and given up treating him as such. Naturally, I thought, from all I had heard and seen, that you had ceased to care for him."

Instead of resenting them, her home-truths seemed to touch the old man.

"I suppose you are right in some sort, Rachel Challice. Though, as I said last night, I don't see that you have acted any better by your sister than I have by my son. But let bygones be bygones on that score. I want to know more about David. Was he ill long?"

"I found him very ill when I came here yesterday evening. He suffered terribly, almost to the end. But all was done for him that was possible. You may make your mind easy about that. He spoke of you——"

"About me," interrupted the old man eagerly. "Yes, go on. What did he say? Did he want to see me?"

"He said he should have liked it, if there had been time. But it all seemed to come so suddenly, and he was so worn out with pain I did not urge it. You could have done no good; while I did not know whether, even if we sent for you, you would come."

"I think I should," he replied shortly. "Go on."

"There is not much more to tell you. I was to say he was sorry to have gone against your wishes. The chief thing he charged me to ask you was if you would be kind to the children for his sake."

"I haven't ever seen them, so it wouldn't be for theirs," said Elias roughly. He felt his only son's death keenly, and his own unjust, harsh conduct yet more sharply. But to show his grief, even before Rachel, was what his pride would not permit.

"It is not too late to help *them*," urged Rachel. "It is the least you can do, Mr. Stephens. I do not ask you to do anything for my poor sister; it is not my place to urge her claim upon you. But for David's children I ask it almost as a right."

"You have plenty of pluck, Rachel Challice," observed old Stephens, looking admiringly at the girl. She was the only person, except his hated enemy, the vicar of Aberclwyn, who was not afraid of speaking plainly to him; and he appreciated it from Rachel as much as he resented it from Mr. Jones.

"I don't know but what I like you the better for it, though," he went on slowly and critically. "It's a pity that sister of yours hasn't a little more of your common sense and go about her. What is she doing now?"

"We will leave Esther out of the discussion," replied Rachel, coldly. "My sister will be my charge. I have no wish to force her, as an unwelcome burden, upon anyone's charity."

"No, you'd rather starve together, and keep your pride. Eh, is that what you mean? Well, pride, though it may be a good thing in its way, is cold comfort at the best of times. I don't suppose you are over and above well off yourself, are you?"

"At all events, my affairs are no concern of yours, Mr. Stephens," interposed Rachel hotly.

"No, my dear, no more they are; you've no claim on me whatever. But my son's widow and my grandchildren have; and I'm not going to shirk the responsibility in either case."

His sharp tone had softened, and something next door to tears glistened in his hard old eyes.

It takes a long time to melt iron; but when once it is softened it is wonderfully pliable.

"I am very glad to hear it," said Rachel briefly.

She was quick enough to perceive that thanks and effusive acknowledgments would not be acceptable in this case. Gratitude in deeds, not words, was what old Elias valued most; and he liked it shown by total submission to his wishes.

"I'd like to see my boy," he said wistfully.

And Rachel took him upstairs into the quiet room, where the still, shrouded form of his dead boy lay.

She went out and closed the door behind her, leaving the old man alone with his dead; while she went back to Esther and the children.

"Come, Essie," she said, lifting the whining, fretting child in her arms. "We will leave the baby with mother, while you and I go down and get breakfast ready."

Little Essie stopped crying, and held out her arms willingly towards Rachel. She had taken a wonderful fancy to this new aunt; and Rachel had that happy knack which attracts children almost at first sight; her management of them, for one so inexperienced, was wonderful; it seemed to come to her naturally.

"This is your eldest grandchild," said Rachel, as Elias came downstairs from the quiet room with its silent occupant.

"She's like her mother," he replied shortly, turning away from the little girl; who set up a howl of terror at sight of the strange, grim-visaged old man.

"Hush, Essie, you will wake baby. Go to your grandfather, dear, and shake hands with him," said Rachel, desperately; and Essie, wonderful to relate, for once did as she was bid, and went towards Mr. Stephens.

"She is obedient at any rate; and that is the first duty of youth," observed Elias approvingly.

Then, as Essie, even exceeding her instructions, not only held out her little plump hand, but raised her pretty pink and white face to his, he stooped down, and kissed the child on her forehead. He looked very red and ashamed of himself when he had done it, and asked Rachel in crosser tones than ever, "What had become of his stick; where had she put it?"

She handed it to him silently from where he had laid it before going upstairs; and then, holding Essie by the hand, she turned to open the door.

As he passed her he thrust a little packet into her hand.

"Keep it," he said gruffly; "and when that's gone come to me for more. Don't let there be any grudging or skimping. I want everything done as well as it can be."

Then he walked quickly away up the road towards Aberelwyn, leaving Rachel no time for thanks, or questions, or acknowledgments of any kind.

Rachel opened the little roll, and found four five-pound notes. Old Elias was not quite so black as she had painted him. She felt a little qualm of conscience as she looked at the crisp bits of paper, and resolved that for the future she would not be quite so harsh and quick in her judgments of other people.

"I ought to be the last to judge anyone," she thought sadly. "I, who have been so misjudged myself. But *he* knows now, whatever he may have once thought. He did not die misdoubting me."

*(To be continued.)*

